# A New Kind of Academic MLP: Addressing Clients’ Criminal Legal Needs to Promote Health Justice and Reduce Mass Incarceration

**DOI:** 10.1017/jme.2024.3

**Published:** 2023

**Authors:** Nicolas Streltzov, Ella van Deventer, Rahul Vanjani, Elizabeth Tobin-Tyler

**Affiliations:** 1: THE WARREN ALPERT MEDICAL SCHOOL OF BROWN UNIVERSITY, PROVIDENCE, RI, USA; 2: BROWN UNIVERSITY SCHOOL OF PUBLIC HEALTH, PROVIDENCE, RI, USA.

**Keywords:** Medical-Legal Partnerships, Incarceration, Criminal Legal Needs, Health Justice

## Abstract

This article describes a new type of medical-legal partnership (MLP) that targets the health and justice concerns of people enmeshed in the U.S criminal justice system: a partnership between clinicians who care for people with criminal system involvement and public defenders. This partnership offers an opportunity to not only improve patient health outcomes but also to facilitate less punitive court dispositions, such as jointly advocating for community-based rehabilitation and treatment rather than incarceration.

*Ms. C is a 42-year-old woman who was recently discharged from the hospital with a diagnosis of a potentially life-threatening autoimmune disorder of her heart. In recent years, she had experienced intermittent street homelessness. Ms. C also has a history of criminal-legal involvement that includes a standing warrant for her arrest secondary to unpaid court fines and failure to present in court. Shortly after discharge from the hospital, Ms. C shared her motivation to engage in treatment for her autoimmune disorder. She stopped smoking, was in recovery for a substance use disorder, maintained a cardiac diet, and worked on mobility and exercise each day. Despite this initial momentum, her criminal-legal status limited her ability to follow treatment recommendations. She avoided public spaces, such as grocery stores and public transportation, for fear of an incidental encounter with police that could lead to arrest. The outstanding warrant also made her ineligible for local homeless shelters, many of which had a policy of notifying police when a potential resident was found to have an active arrest warrant.*

*Given her need for prompt diagnostic workup and treatment, it was clear that incarceration would pose a serious threat to her health, as would another episode of street homelessness. With permission from Ms. C, her care team contacted the local public defender’s office to discuss her criminal-legal status with an attorney. The care team wrote a letter describing the worrisome medical implications of both incarceration and the continued possibility of arrest for Ms. C. The public defender, along with the medical student on the care team, accompanied Ms. C. to court and presented the letter to the judge. The judge agreed with the interdisciplinary team’s arguments, cleared the charges against her, and waived the outstanding court fines and fees. Ms. C was able to safely access a nearby shelter for housing as she engaged with social service agencies and proceeded with her medical treatment.*

This essay explores a new type of medical-legal partnership (MLP) that targets the health and justice concerns of people enmeshed in the U.S criminal justice system: a partnership between clinicians who care for people with criminal system involvement and public defenders. This offers an opportunity to not only improve client health outcomes but also to facilitate less punitive court dispositions.

## Introduction

The United States incarcerates more individuals per capita than any other country in the world. Currently, there are nearly 2 million people in the U.S. living in a variety of correctional facilities, including state and federal prisons, local jails, juvenile facilities, immigrant detention centers, Indian country jails, and state psychiatric hospitals. Further, the carceral ecosystem in the U.S. extends beyond these residential facilities — nearly 4 million people live under community supervision (probation or parole).^1^ During the COVID-19 pandemic, the U.S experienced a drop in jail and prison populations that provided some hope that the U.S. approach to punishment would begin to more closely resemble that of other countries. However, data have since made clear that these reductions were unintended results of court slowdowns as opposed to deliberate policy actions to decarcerate.^2^ As a result, the jail and prison populations are once again approaching pre-pandemic levels.^3^

Incarceration has a profound impact on individual and population health, which drives significant racial and socioeconomic health inequities, especially for low-income Black Americans. Low-income people with chronic disease, who have experienced homelessness, and who struggle with substance use disorders and/or mental health problems face a high likelihood of carceral system involvement.^4^ As described above in the case of Ms. C, even the threat of incarceration can have overwhelming consequences for an individual with a fragile health trajectory. Healthcare providers who care for clients with carceral system involvement often experience significant frustration as they witness the effects of that system on their clients’ health. Similar frustrations are often felt by public defenders who may struggle with how best to present their clients’ medical history in court to demonstrate the health implications of punitive court dispositions, including incarceration.

This essay explores a new type of medical-legal partnership (MLP) that targets the health and justice concerns of people enmeshed in the U.S criminal justice system: a partnership between clinicians who care for people with criminal system involvement and public defenders. This offers an opportunity to not only improve client health outcomes but also to facilitate less punitive court dispositions. First, we highlight how partnering clinicians and public defenders draws on the typical civil legal MLP model and how it diverges by explicitly focusing on criminal defense. Next, we describe the goals and framework of the partnership between the Lifespan Transitions Clinic and the Rhode Island Public Defender’s Office in Providence, Rhode Island. Finally, we discuss how an MLP focused on criminal law serves as an excellent interprofessional education and training opportunity for future legal and health care practitioners interested in mass incarceration and health justice.

## Health and Incarceration

Socioeconomic status plays a central role in mass incarceration. The carceral population is disproportionately poor compared to the overall U.S. population.^5^ In addition, the criminal legal system is marked by stark racial disparities. For instance, Black Americans make up 38% of the incarcerated population despite representing only 12% of U.S. residents.^6^ Race is a central factor for two reasons: 1) people of color experience greater rates of poverty than white people, and 2) structural racism is deeply embedded in the U.S. policing and judicial systems.^7^ The criminal legal system compounds poverty by setting bail, imposing supervision fines, rendering court debt, and decimating future job opportunities, which are just a few of the many collateral consequences of being incarcerated in this country.^8^

Carceral exposure also has a significant negative impact on the health and well-being of formerly incarcerated individuals and their surrounding communities, with both direct and indirect effects. Incarceration and community supervision directly influence health by interrupting treatment, forcing numerous transitions of care, causing stress which impacts cardiovascular and other chronic conditions, and removing autonomy and a sense of control over life, among other factors.^9^ The indirect effects relate to the social and structural determinants of health — incarceration and community supervision limit access to employment, business and occupational licensing, public benefits, housing vouchers, shelter access, and voting rights, to name a few.^10^ There is robust evidence demonstrating that lack of access to these social resources correlates with poor health.^11^ Indeed, issues of economic, racial, and health justice converge at the intersection of incarceration and health.

## Expanding the MLP Approach to Address Criminal Legal Needs

MLPs aim to assist low-income and marginalized client populations with health-harming legal needs and improve their overall health by addressing social and structural determinants of health and improving access to justice.^12^ Primary care is an ideal setting for MLP integration, particularly in practices that emphasize a team-based approach to client care.^13^ Early models have demonstrated improvements in client adherence to clinical regimens, reduced emergency department visits, and a number of other benefits.^14^

MLPs are a powerful tool for health justice because of their ability to detect, document, and address injustices that clients experience.^15^ Typically, MLPs seek to address unmet *civil l*egal needs and have not historically addressed *criminal l*aw matters. For example, while MLP screening tools include the role of legal status as a social determinant of health, the questions tend to center the collateral consequences of a client’s criminal record, not current criminal legal matters affecting a client’s health and well-being.^16^

A number of MLPs specifically target the civil legal needs of people with a criminal history, most often by partnering with a transitions clinic (a medical clinic specifically focused on serving people returning from incarceration) with a legal aid organization or law school clinic to serve people who are reentering their communities after serving time in prison.^17^ These MLPs, such as the New Haven Transitions MLP, serve as a “one-stop shop” to help clients navigate the vast collateral consequences associated with a criminal record, seek expungement of criminal records that serve as barriers to reentry, and maintain access to health care and other vital social services. Outside of the select transitions MLPs, most MLPs are not designed to assist clients with needs related to criminal-legal disposition or ongoing carceral control. Over the past 30 years, the number of Americans under community supervision (probation/parole) has skyrocketed. There are currently 2 million more people on probation than in 1980, and the probation system makes up the greatest proportion of the largest correctional population in the world.^18^ Technical probation violations and unpaid court fines and fees keep millions of Americans ensnared within the criminal justice system whether through direct supervision or servicing of debts. Given the immediate and down-stream health impacts of criminal-legal debts and excessive use of probation and incarceration in the U.S., MLPs can serve as a vital intervention to prevent or reduce criminal system involvement, while also addressing social and structural determinants of health.^19^

## Integrating Criminal Legal Needs into the Medical-Legal Partnership Paradigm

Unlike in civil legal matters, individuals facing criminal legal charges are guaranteed the right to an attorney regardless of their ability to pay.^20^ The right to counsel in state criminal cases is achieved through state-funded public defender programs. A partnership of health care organizations and public defenders offers a unique opportunity to address both individual and systemic health and justice issues. Medical evidence and information about a client’s health and social context can be invaluable for public defenders as they investigate and advocate for their clients.

Increasingly, clinicians are realizing how the ripple effects of mass incarceration impact their clients’ health and well-being. For instance, studies of clients receiving primary care in urban areas have shown that up to 50% have been arrested, incarcerated, or have a family member who has been arrested or incarcerated.^21^ As a result, our group has recently advocated to incorporate questions about involvement in correctional control into standard social determinants of health screening questionnaires in clinical settings.^22^ Screening for any social determinant of health, including correctional control, is best done using a team-based approach such as an MLP, where there are professionals with the expertise to respond to unmet needs. Public defenders bring valuable knowledge about the criminal justice system and how to employ medical evidence and information about clients’ health and social context in court to achieve better outcomes.

## Developing a Medical-Legal Partnership Focused on Clients’ Criminal Legal Needs in Rhode Island

Established in 2018, the Lifespan Transitions Clinic (LTC), located at the Rhode Island Hospital Center for Primary Care (CPC), provides services to clients with carceral system involvement. CPC is an academic residency clinic that serves as an education and training site for medical students and residents from the Warren Alpert Medical School of Brown University. LTC is part of the national Transitions Clinic Network.^23^ The Rhode Island Public Defender’s Office (RIPD) provides holistic advocacy to its clients, including through its Social Services Unit, which partners social workers with lawyers to address the social and legal needs of clients. In 2019, as LTC clinicians became increasingly frustrated by the role of incarceration, re-incarceration and community supervision in their clients’ lives, its leadership sought out collaboration with RIPD, recognizing the two organizations likely served many of the same people.

LTC clinicians sought to advocate for their clients, but they struggled to navigate a complex and confusing criminal legal system. Similarly, RIPD frequently reached out to their clients’ medical providers, but often experienced difficulty establishing contact or sustaining long-term relationships with providers. RIPD attorneys and social workers, therefore, struggled to understand the medical conditions their clients self-reported and the implications of these conditions for their cases. Clinicians from LTC and staff from RIPD’s Social Services Unit met to brainstorm the potential for developing a partnership. Below, we highlight some of the key steps that led to the establishment of the formal LTC-RIPD MLP.

## Relationship-Building and Strategies for Communication

The first and often most important step in starting a new MLP is to develop a trusting relationship between the partners through the establishment of shared goals. These goals include: 1) enabling seamless and ethical communication between LTC and RIPD staff; 2) making available clinical and social context to judges presiding over criminal cases involving LTC clients; and, 3) protecting LTC clinicians against developing a dual loyalty (i.e. simultaneously trying to balance the health care of a client and the safety of the community) by relying on RIPD staff to determine when and how LTC clinicians should provide information for the judicial system.

As described above, both LTC and RIPD staff were excited to improve their ability to serve their clients by seeking out the expertise of the other and developing a mechanism for regular communication and collaboration. LTC and RIPD developed concrete mechanisms for ongoing communication so that the momentum would not be lost and established a single point of contact at LTC and at RIPD who would serve as the liaison for communication about clients. This avoided confusion for busy staff at both organizations about who to call about shared clients.

## Screening Clients for Criminal System Involvement

Next, LTC created specific workflows for identifying clients who could benefit from the partnership with RIPD. LTC employs community health workers (CHWs) who are individuals with lived experience of incarceration who connect clients to community resources and help them navigate the medical system. The CHWs screen clients to determine if they have current involvement in the criminal legal system. Once identified, clients are asked for their consent for direct communication between their medical provider and their public defender about their medical issues and their criminal case. LTC and RIPD developed a custom release of information form which is offered to all clients at their first primary care visit to promote proactive, as opposed to reactive, communication between LTC and RIPD.

**Figure 1:**
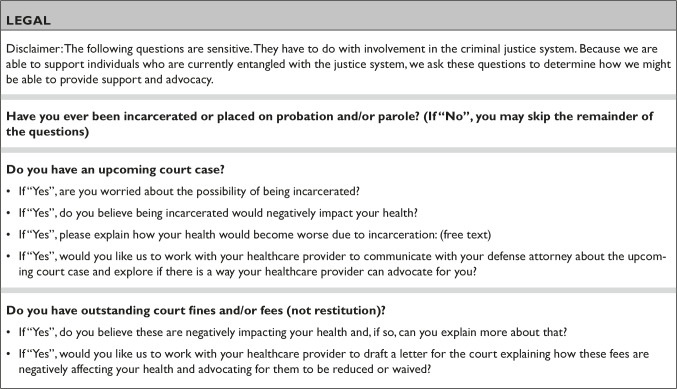
Sample set of criminal justice involvement screening questions

Primary care, with its focus on prevention and longitudinal clinician-client relationships, is an appropriate setting for identification of criminal-legal issues that implicate health. Since many primary care settings already employ screening tools such as the PHQ-9 for depression and social determinants of health questionnaires, adding questions about carceral involvement deepens providers’ understanding of clients’ health, social, and legal needs. Given the power dynamic that can exist between clinicians and clients as well as the elevated rates of PTSD and trauma-exposure among people in carceral settings, permission must be acquired before screening and screening must be conducted using a trauma-informed approach.^24^ This requires sensitivity to potential triggers for traumatic memories, attempting to minimize the risk of re-traumatization, and avoiding dynamics that could replicate the traumatic setting.^25^ LTC staff preface screening by explaining that because there is robust evidence demonstrating that carceral system involvement can be harmful to health, LTC screens all clients to determine how this involvement may affect health and to identify client supports. A sample set of criminal justice involvement screening questions are included in Figure 1.

## Developing the Client’s Medical-Legal Team to Advocate in Court

When a client screens positive for an upcoming criminal court case, their community health worker and/or primary care physician asks two basic follow up questions: first, how this new criminal legal issue is impacting their medical and/or mental health and second, if incarcerated, how that might impact their health. This self-reported information is then paired with objective data about the client’s clinical history to help develop the LTC team’s understanding of the relationship between the client’s criminal justice involvement and their overall health and well-being. Second, LTC staff ask permission to speak with the public defender’s office to discuss the client’s case and determine whether there is a way in which the medical team can offer support. Ideally, the release of information allowing the LTC team to discuss the client with RIPD is already on file from the first primary care visit. The benefit of obtaining a release of information at the first visit is that communication between the medical and legal teams can proceed even when the client is not present. For instance, the public defender may contact the medical team if a client misses a court date. Similarly, the medical team may contact the public defender if they learn that a client has been incarcerated.

The medical team then communicates directly with the public defender and provides medical information relevant to the client’s legal case. At this point, RIPD staff decide whether the medical team should detail this information for the court, based on factors including the specific charge and the client’s history. That this decision is made by RIPD, and not the medical team, is vital in that it protects the medical team from inadvertently engaging in a dual loyalty; the focus of the medical team is solely on the client’s health and well-being. In the vast majority of cases, RIPD requests documentation from LTC staff to be used in court. Specifically, clinicians may document their concerns about a client’s health as related to the burden associated with court fines and fees. LTC has developed customizable form letters for this purpose that address, for example: 1) the impact incarceration might have on the health of a client and 2) the relationship between the alleged crime and a client’s medical history. The partnership regularly conducts trainings for team members — community health workers, physicians and public defenders, and other RIPD staff — on all aspects of the workflow, including communication, screening, and use of clinician letters for court advocacy.

## Navigating Ethical Dilemmas

A prominent challenge for MLPs is navigating ethical issues related to interprofessional advocacy. LTC and RIPD acknowledged that the ethical issues inherent in MLPs would be heightened in a MLP focusing solely on criminal legal needs. Ethical dilemmas include whether client permission is required for all future disclosures between LTC and RIPD after a client provides for release of information at an initial visit; medical providers’ ethical obligations as mandated reporters of child and elder abuse while navigating the criminal-legal space; and decision-making by a medical provider who believes that release from jail time would increase a client’s risk of danger to self or others.

A prominent challenge for MLPs is navigating ethical issues related to interprofessional advocacy. LTC and RIPD acknowledged that the ethical issues inherent in MLPs would be heightened in a MLP focusing solely on criminal legal needs. Ethical dilemmas include whether client permission is required for all future disclosures between LTC and RIPD after a client provides for release of information at an initial visit; medical providers’ ethical obligations as mandated reporters of child and elder abuse while navigating the criminal-legal space; and decision-making by a medical provider who believes that release from jail time would increase a client’s risk of danger to self or others.

The latter situation typically arises not because a correctional facility is truly the most appropriate destination for the client, but rather because of a dearth of other available resources. For example, the client could be an elderly individual with a history of severe traumatic brain injury who is in a wheelchair. The client lives on the street, having been evicted from several apartments and skilled nursing facilities due to impulsive behaviors. Numerous medications, including mood stabilizers and antipsychotics, have not mitigated these episodes. He is typically found on the street, where he is soiled, unable to move, and tends to articulate suicidal thoughts. He is taken to the emergency department many times a week, but psychiatric intervention does not have a lasting impact and neuropsychiatric evaluation deems him to have capacity, precluding certification. He does not qualify for a group home due to lack of a developmental/intellectual disability diagnosis as a youth and the state’s hospital is underfunded and unable to accept more clients. He has outstanding warrants and one day he is detained and subsequently brought to court. After extensive interprofessional discussion and consultation with other professionals in the state, the LTC-RIPD team informs the judge of the situation and names the reality that, at this moment, prison is the safest option for the client and the de facto safety net in society for a client in this situation.

To address ethical challenges such as these and the one presented by the hypothetical example above, the LTC-RIPD team developed a framework for navigating challenging situations that entails interprofessional consultation, interdisciplinary case conferences, and ongoing professional development and training. By taking each situation on a case-by-case basis, the collaborative framework allows for challenging decisions to reflect the ethical priorities that emerge from discussion between a diverse group of empowered team members and professionals. This has helped the team to navigate the delicate balance between protecting clients’ rights, ensuring holistic care, and fulfilling professional obligations.

## Evaluating the Partnership

**Figure 2:**
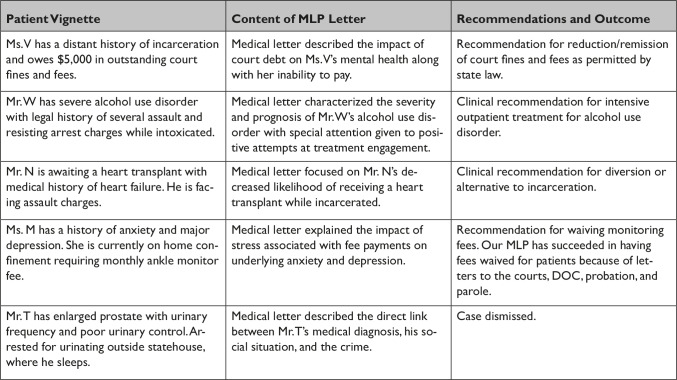
Sample set of collaborative cases by Lifespan Transitions Clinic and Rhode Island Public Defender Partnership

There is ongoing data collection to measure quality outcomes and to quantify services provided. In its first year of operation, LTC served over 70 clients with a nearly 90% show rate for clinic appointments, and each client received an average of four appointments per year, allowing multiple opportunities to assess and intervene in changes to criminal legal needs.^26^ In total, LTC and RIPD have collaborated on approximately 85 unique cases since 2018, and the influence of the partnership on judicial decision-making and sentencing has been detailed in local press.^27^ In numerous instances, judges reported being moved by the medical reports to reduce or completely spare incarceration due to consideration of the individual’s health. A sample set of the types of cases on which LTC and RIPD have collaborated are included in Figure 2.

## Training and Education in the Criminal Justice MLP

As noted earlier, the LTC-RIPD MLP is embedded in an academic residency clinic affiliated with the Alpert Medical School of Brown University and serves as a training site for both medical students and internal medicine residents. The MLP also serves as a site for law students from Roger Williams University School of Law (RWU Law) to provide pro bono legal support to the partnership. The LTC-RIPD partnership not only provides important services and supports to low-income and marginalized client populations; it also contributes to systems change by training the next generation of physicians and lawyers to understand the role of carceral system involvement in health, to develop interprofessional problem-solving skills, and to advocate for clients who are often stigmatized and disconnected from support.

Medical trainees benefit from a better understanding of the criminal legal system that shapes their clients’ lives and from development of advocacy skills that are often not otherwise taught. Firsthand exposure to the legal, social, and health challenges experienced by clients with carceral system involvement provides students and residents with invaluable knowledge and skills.^28^ Medical trainees work closely with clients, draft physician letters about a client’s health needs and the potential effect of reincarceration on their health, and accompany clients to court. For example, in assisting with Ms. C’s case, highlighted above, medical students built relationships with public defenders and other community members, contributed to the physician letter presented to the judge, and ultimately improved the material conditions of Ms. C’s life. This experience expanded their own imagination for what can be achieved in clinical practice.

The LTC-RIPD MLP also serves as a learning opportunity for law students through the Pro Bono Collaborative at RWU Law. At LTC, law students with legal supervision from faculty develop and regularly update physician form letters used by the MLP. These form letters are stored in “Docs for Health,” an online resource available to LTC clinicians. Docs for Health include letters on a wide range of issues, including civil legal and social needs such as housing, immigration, transportation, and criminal justice-related topics that are relevant to client health.

Similar to medical trainees, law students gain early exposure to and intimate knowledge of tangible ways to advocate for clients with criminal justice system involvement through MLP. They learn to advise physicians on how to document and present medical evidence in a legally compelling way and how to navigate changes in the law that affect clients’ rights and health. Again, this knowledge during their training instills the value of partnering with the medical community in their future practice of law.

## Conclusion

Given the profound impact of the U.S. criminal justice system on health, particularly for low-income people of color and other marginalized populations, expanding the MLP approach to address clients’ criminal legal needs is an important step in MLP evolution. The LTC-RIPD MLP demonstrates some of the benefits of such a partnership for clients. As an academic MLP it offers the potential for training future medical and legal practitioners about the role of mass incarceration in health and opportunities for interprofessional collaboration and advocacy. Until the U.S. begins to sufficiently address its penchant for punitive approaches to a wide range of deeply entrenched social and health problems, this type of interprofessional training and practice is a promising approach to reducing the harms of mass incarceration for individuals, families, and communities.

## Note

The authors have no conflicts to disclose.
